# Analysis of the differentially expressed genes in the combs and testes of Qingyuan partridge roosters at different developmental stages

**DOI:** 10.1186/s12864-024-09960-2

**Published:** 2024-01-04

**Authors:** Hao Qi, Zhidan Deng, Fei Ye, Junwei Gou, Miaoxin Huang, Hai Xiang, Hua Li

**Affiliations:** Guangdong Provincial Key Laboratory of Animal Molecular Design and Precise Breeding, College of Life Science and Engineering, Foshan University, 528225 Foshan, Guangdong China

**Keywords:** Chicken, Comb, Testis, Sexual maturity, Weighted correlation network analysis (WGCNA)

## Abstract

**Background:**

The sexual maturity of chickens is an important economic trait, and the breeding of precocious and delayed puberty roosters is an important selection strategy for broilers. The comb serves as an important secondary sexual characteristic of roosters and determines their sexual precocity. Moreover, comb development is closely associated with gonad development in roosters. However, the underlying molecular mechanism regulating the sexual maturity of roosters has not yet been fully explored.

**Results:**

In order to identify the genes related to precocious puberty in Qingyuan partridge roosters, and based on the synchrony of testis and combs development, combined with histological observation and RNA-seq method, the developmental status and gene expression profile of combs and testis were obtained. The results showed that during the early growth and development period (77 days of age), the development of combs and testis was significant in the high comb (H) group versus the low comb (L) group (*p* < 0.05); however, the morphological characteristic of the comb and testicular tissues converged during the late growth and development period (112 days of age) in the H and L groups. Based on these results, RNA-sequencing analysis was performed on the comb and testis tissues of the 77 and 112 days old Qingyuan Partridge roosters with different comb height traits. GO and KEGG analysis enrichment analysis showed that the differentially expressed genes were primarily enriched in MAPK signaling, VEGF signaling, and retinol metabolism pathways. Moreover, weighted correlation network analysis and module co-expression network analysis identified *WNT6, AMH, IHH, STT3A, PEX16, KPNA7, CATHL2, ROR2, PAMR1, WISP2, IL17REL, NDRG4, CYP26B1*, and *CRHBP* as the key genes associated with the regulation of precocity and delayed puberty in Qingyuan Partridge roosters.

**Conclusions:**

In summary, we identified the key regulatory genes of sexual precocity in roosters, which provide a theoretical basis for understanding the developmental differences between precocious and delayed puberty in roosters.

**Supplementary Information:**

The online version contains supplementary material available at 10.1186/s12864-024-09960-2.

## Background

In animals, sexual maturation is accompanied by aging, changes in tissue morphology and an increase in reproductive capacity [1–3]. Sexual maturity of the chickens is an important economic trait, and the breeding of precocious or delayed puberty individuals is an important selection strategy for broilers. Economically, the reproductive ability of roosters is more important than that of hens, in a breeding flock, as the roosters can fertilize many eggs [4]. In general, the first crow of the rooster is considered an indicator of its sexual maturity [5]. The primary reason for the occurrence of precocious puberty among roosters is the premature activation of the hypothalamic-pituitary-gonadal axis and the secretion of multiple sex hormones, which lead to the development of secondary sexual characteristics [6]. With multiple generations of breeding, the age of mating advanced and the comb size increased in the precocious puberty roosters, leading to many economic benefits [7, 8]. Qingyuan Partridge chicken is a renowned high-quality chicken breed that is well-known for its delicious meat in China and neighboring countries [9]. To meet the market’s demand, it is essential to breed with exceptional growth performance and early maturity of Qingyuan Partridge roosters. Therefore, it is necessary to analyze the underlying molecular mechanism leading to precocious puberty in roosters.

The comb is an important secondary sexual characteristic of the roosters and serves as an indicator of the reproductive capacity and sexual precocity of chickens [10], thus it can be used to determine the sexual maturity of roosters for broiler production.Testes are the primary reproductive organ of male animals, and studies have shown an association between testis weight and sexual maturity [4]. Moreover, studies have shown that comb development is closely associated with gonad development in roosters and that testes weight is correlated with comb development in roosters aged < 98 days [11]. A study found that roosters with larger combs typically have higher testicular weights, semen production, and androgen levels [12]. Previous studies have shown that the genes Hydroxyacid Oxidase 1 (*HAO1*), bone morphogenetic protein-2 (*BMP2*), SRY-box transcription factor 5 (*SOX5)*, and androgen receptor (*AR*) affect comb morphology and development [13–16]; however, their roles in the regulation of sexual precocity of Qingyuan Partridge roosters remain to be studied.

In chickens, sexual maturation is regulated by a complex network of genes [17]; however, studies on precocious puberty in roosters are limited compared to those in hens. A study found that an allele in the comb trait locus that leads to an increase in comb weight delays the initiation of sexual maturity in chickens [18]. In addition, studies have found that GTPase-activating Rap/Ran-GAP domain-like 1(*GARNL1*) [19], Dehydrogenase/Reductase (SDR family)Member 12 (*DHRS12*) [20], and G-protein coupled receptor 39 (*GPR39*) [21] are positively correlated, while go-nadotropin-releasing hormone-I (*GnRH-I*) [22] is negatively correlated with the egg laying age and ovarian development in hens. Moreover, a study found that Transcription factor 21 (*TCF21*) [23] and gamma-aminobutyric acid A receptor alpha 1 (*GABRA1*) [21] can stimulate testicular growth and development in roosters. However, the underlying molecular mechanism associated with the onset of sexual precocity in roosters remains unclear, necessitating further studies on the network of genetic factors that lead to this trait.

In this study, we used RNA-sequencing (RNA-seq) analysis to investigate the differential gene expression in the combs and testis of roosters with different comb heights. Thereafter, we identified key regulatory genes associated with precocious puberty and delayed puberty in roosters, which may provide a theoretical basis to improve the breeding efficiency of high-quality chickens and reduce the cost of breeding.

## Result

### Morphological differences in the comb and testes of Qingyuan partridge roosters at different developmental stages

**Fig. 1:**
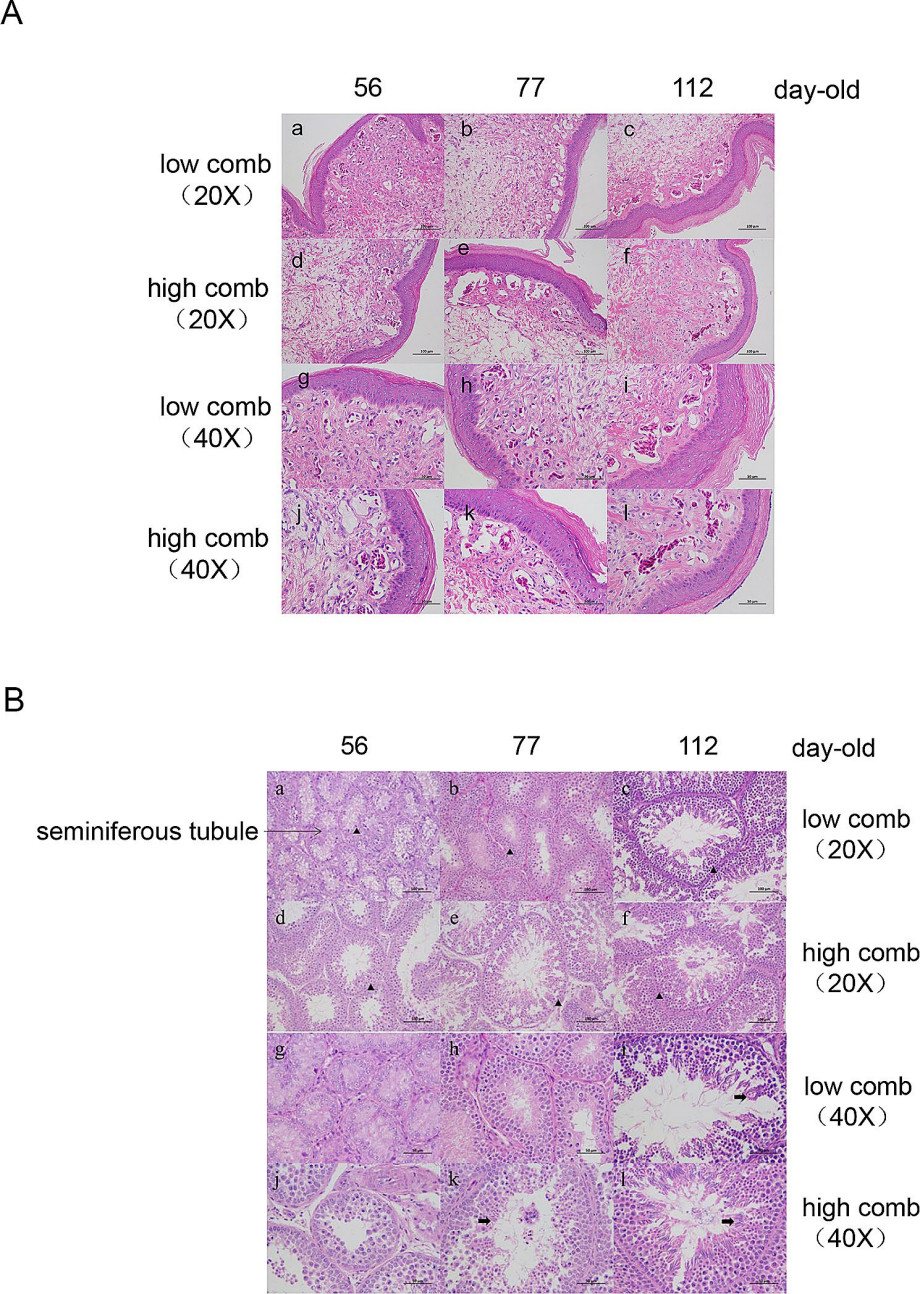
Morphological differences in the comb and testis tissues of the Qingyuan partridge roosters at different developmental stages. (**A** and **B**) Morphological analysis of the comb (**A**) and testis (**B**) tissues of the high-and low-comb groups at different developmental stages using hematoxylin and eosin staining. Original magnification: **a**-**f** in Figure **A** and **B**: 20x, scale bar = 100 μm and g-l in Figure **A** and **B**: 40x, scale bar = 50 μm. In Figure **B**, ▲ indicates spermatogonium and → indicates sperm cells

This study demonstrated that the H group of Qingyuan Partridge roosters exhibited significantly higher testicular weight and testicular index during the rapid growth period (56 days of age) and early growth and development period (77 days of age) compared to the L group. However, no statistically significant difference was observed between these two parameters in the later stages of development (112 days of age) (Table 1). HE staining of the comb tissues (Fig. 1A) showed that the capillaries in the comb expanded, the collagen fibers became sparse, and the number of fibroblasts decreased with an increase in age. In addition, significant changes were observed in the comb dermis during 56 and 77 days of age. Comparison the comb tissues at the same developmental stage revealed that the capillaries in the comb tissues expanded earlier, and the collagen fibers decreased much earlier in the H group compared to the L group. However, the morphological characteristic changes in the comb tissues gradually converged between the H group and the L group in 112 days of age.

**Table 1:** Significance analysis of the correlated characteristics between Qingyuan partridge roosters at different age stages

Trait	56-day-old		77-day-old		112-day-old	
	H (n = 15)	L (n = 15)	H (n = 15)	L (n = 15)	H (n = 15)	L (n = 15)
body weight (g)	656.3 ± 35.7	652.7 ± 83.6	1089.5 ± 79.3	1075.2 ± 67.7	1421.7 ± 96.8	1455 ± 81.2
comb height (mm)	32.04 ± 4.99^A^	21.52 ± 2.74^B^	43.79 ± 3.95^A^	36.50 ± 4.66^B^	59.84 ± 5.36	56.89 ± 5.92
weight of left testis (g)	0.81 ± 0.54 ^A^	0.08 ± 0.023 ^B^	4.85 ± 1.67 ^A^	2.08 ± 1.89 ^B^	9.35 ± 2.1	8.48 ± 3.0
area of left testis (mm^2^)	0.83 ± 0.54 ^A^	0.15 ± 0.15 ^B^	437.6 ± 100.3 ^A^	237.95 ± 163.2 ^B^	812.63 ± 109.29	751.7 ± 168.97
weight of right testis (g)	0.7 ± 0.45 ^A^	0.07 ± 0.02 ^B^	4.07 ± 1.45 ^A^	1.76 ± 1.63 ^B^	8.15 ± 2.73	7.28 ± 2.99
area of right testis (mm^2^)	0.41 ± 0.38 ^A^	0.13 ± 0.15 ^B^	331.99 ± 82.7 ^A^	196.4 ± 133.5 ^B^	754.03 ± 225.81	671.65 ± 195.21
weight of total testes (g)	1.51 ± 0.98 ^A^	0.15 ± 0.04 ^B^	8.92 ± 3.11 ^A^	3.83 ± 3.52 ^B^	17.5 ± 4.81	15.77 ± 5.9
testis index	0.23 ± 0.15 ^A^	0.02 ± 0.009 ^B^	0.83 ± 0.3 ^A^	0.36 ± 0.33 ^B^	1.23 ± 0.39	1.08 ± 0.37

Note: H: high-comb group; L: Low-comb group. All traits were compared between the high and low comb groups at the same age. Unmarked indicates no significant difference (*P* > 0.05), while capital letters indicate a significant difference (*P* < 0.05)

The diameter of the seminiferous tubules and the number of spermatogenic epithelium cell layers gradually increased with age in testis tissues by HE staining (Fig. 1B).

The testicular development was faster in the H group compared with the L group at 56 and 77 days of age, with the presence of sperm cells observed in the H group at 77 days. Additionally, we have observed that the H group exhibited an earlier onset and greater diameter of the complete testicular seminiferous tubule structure in comparison to the L group. However, the morphological changes in the testis tissues of the H and L groups gradually converged at 112 days.

Altogether, these results suggest that the rapid development of the comb and the testis tissues is synchronous during the early growth and development periods in Qingyuan Partridge roosters. Furthermore, the results reveal that the morphology of the comb and testicular tissues is synchronized between the H and L groups during the late growth and development period. RNA-Seq analysis of the comb and testis tissues in the H and L groups was performed at 77 and 112 days of age, to explore the relationship between the spatiotemporal regulation of the key genes associated with comb and testis development and sexual maturity of roosters.

### DEGs in the comb and testis tissues of the H and L groups at the same developmental stage

**Fig. 2:**
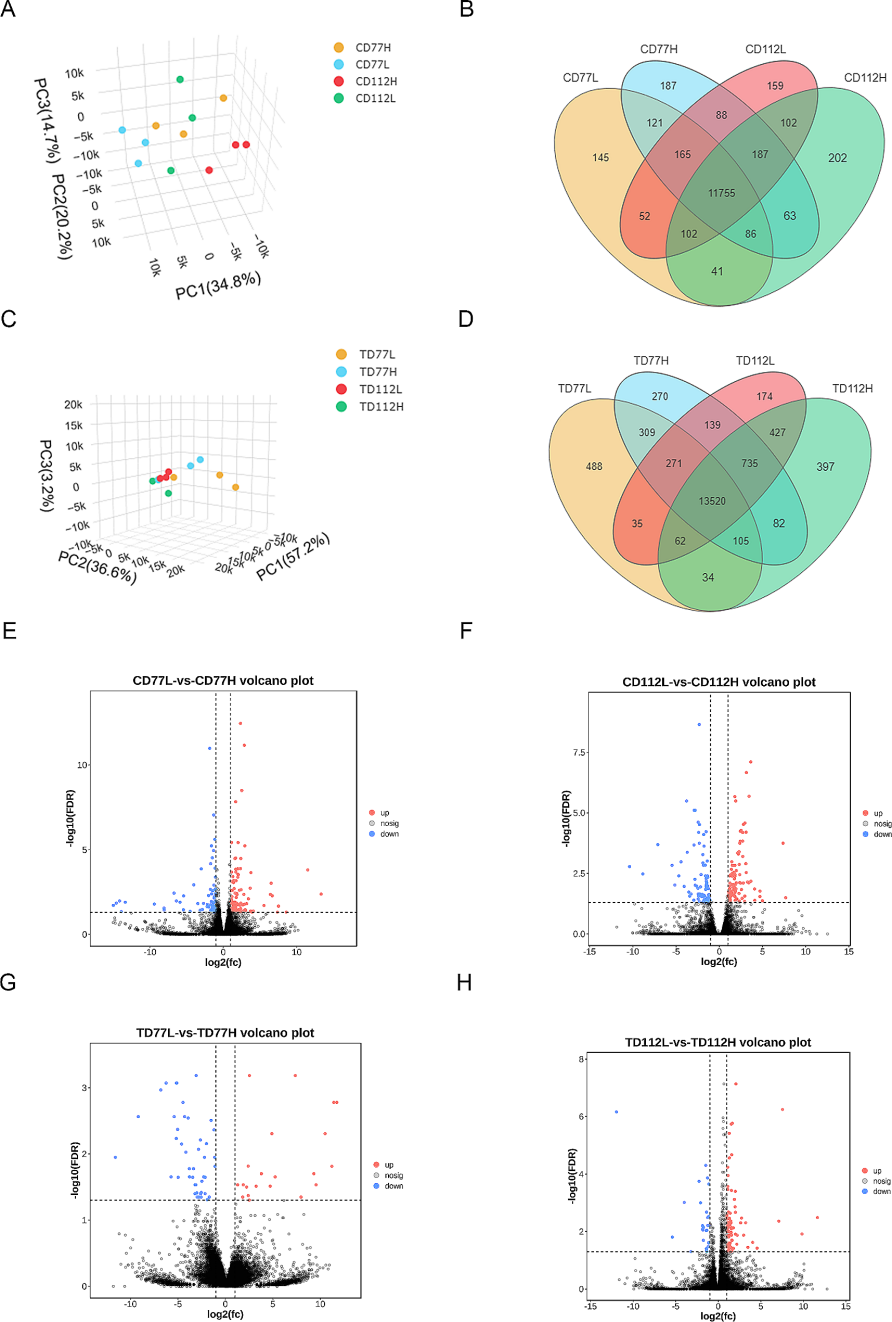
Differentially expressed genes (DEGs) in the comb and testis tissues of the high-and low-comb groups at the same developmental stage. (**A**, **C**) PCA analysis of RNA-Seq data in the comb (**A**) and testis (**C**) tissues; (**B**, **D**) The Wayne map of differentially expressed genes in the comb (**B**) and testis (**D**) tissues; (**E** and **F**) The volcano maps of the DEGs in the comb tissues of the 77 (**E**) and 112 (**F**) day-old high- and low-comb groups and (**G** and **H**) the volcano maps of DEGs in the testis tissues of the 77 (**G**) and 112 (**H**) day-old high- and low-comb groups

A principal component analysis (PCA) was conducted on the fragments per kilobase per million mapped fragments of each comb (Fig. 2A) and testis (Fig. 2C) sample, and it was found that all the subgroups had excellent within-group repeatability and between-group variability. The Wayne diagrams in Fig. 2B and D show the number of DEGs in the comb and testis tissues, respectively. RT-qPCR analysis of the DEGs (n = 6 each) from the comb and testis tissues of the 77 and 112-day-old roosters (Figure S1A-S1D) revealed a consistency between the RNA-seq and gene expression results, thus proving the reliability of the results.

In this study, we identified a total of 222 and 44 DEGs (FDR < 0.05 and|log2FoldChange| >1) in the comb (126 upregulated and 96 downregulated genes; Fig. 2E and S2A) and testis (16 upregulated and 28 downregulated genes; Fig. 2G and S2C) tissues of the 77-days-old H and L group roosters, respectively. In contrast, we identified a total of 185 and 65 DEGs in the comb (93 upregulated and 92 downregulated genes; Fig. 2F and S2B) and testis (39 upregulated and 26 downregulated genes; Fig. 2H and S2D) tissues of the 112-days-old H and L group roosters, respectively.

**Fig. 3:**
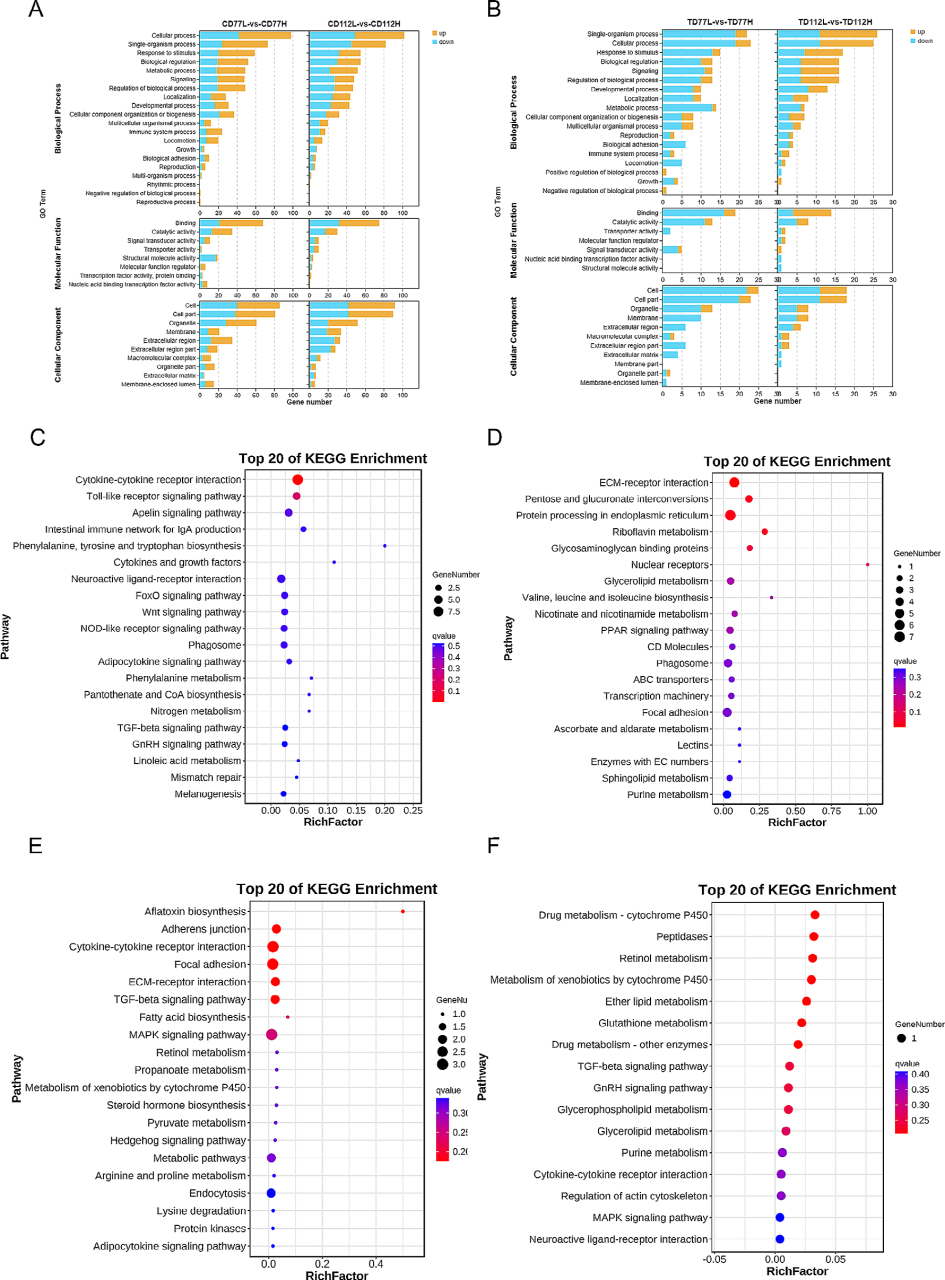
Gene Ontology (GO) and Kyoto Encyclopedia of Genes and Genomes (KEGG) analysis of DEGs in the comb and testes tissues of the high- and low-comb groups at the same developmental stage. (**A** and **B**) GO enrichment analysis of the DEGs in the comb (**A**) and testes (**B**) tissues in the molecular function, cellular composition, and biological processes categories; (**C** and **D**) KEGG enrichment analysis of the DEGs in the comb tissues of the 77 (**C**) and 112 (**D**) day-old high-and low-comb groups; (**E** and **F**) KEGG enrichment analysis of the DEGs in the testes tissues of the 77 (**E**) and 112 (**F**) day-old high-and low-comb groups

These DEGs were subjected to GO and KEGG enrichment analyses to determine their biological functions. GO enrichment analysis showed that the DEGs were significantly enriched in signal-organism processes, cellular processes, response to stimulus, reproduction and developmental process (Fig. 3A and B). KEGG enrichment analysis showed that the DEGs in the comb tissues of the H and L groups at 77 days of age were significantly enriched in the adipocytokine signaling pathway, cytokine-cytokine receptor interaction, and Wnt signaling pathway (Fig. 3C) and that the DEGs in the testis tissues of the H and L groups at 77 days of age were significantly enriched in cytokine-cytokine receptor interaction and the MAPK signaling pathway (Fig. 3E). Furthermore, KEGG enrichment analysis showed that the DEGs in the comb tissues of the H and L groups at 112 days of age were significantly enriched in ECM-receptor interaction, glycosaminoglycan binding proteins, nuclear receptors, and PPAR signaling pathway (Fig. 3D) and that the DEGs in the testis tissues of the H and L groups at 112 days of age were significantly enriched in Drug metabolism-cytochrome P450 and retinol metabolism pathway (Fig. 3F).

Comparative analysis of the DEGs in the comb and testis tissues of the H and L groups at 77 and 112 days of age revealed that secreted frizzled related protein 1 (*SFRP1*), cathelicidin 2 (*CATHL2*), cytochrome P450 family 26 subfamily B member 1 (*CYP26B1*), and *WNT6* were significantly differentially expressed in the comb tissues, while Indian hedgehog signaling molecule (*IHH*), *WNT6*, and receptor tyrosine kinase like orphan receptor 2 (*ROR2*) were significantly differentially expressed in the testis tissues of the H and L groups at 77 days of age, suggesting that these genes may be responsible for the differences in the precocity and delayed puberty in Qingyuan Partridge roosters. Additionally, we found that corticotropin releasing hormone binding protein (*CRHBP*), *AMH*, and HtrA serine peptidase 1 (*HTRA1*) were significantly differentially expressed in the comb tissues, while *CYP26B1* and *AMH* were significantly differentially expressed in testis tissues of the H and L groups at 112 days of age, suggesting that these downregulated genes may be responsible for the compensatory growth during the late developmental stages of the sex late-maturing Qingyuan Partridge roosters.

### DEGs in the comb and testis tissues of the H or L group at different developmental stages

**Fig. 4:**
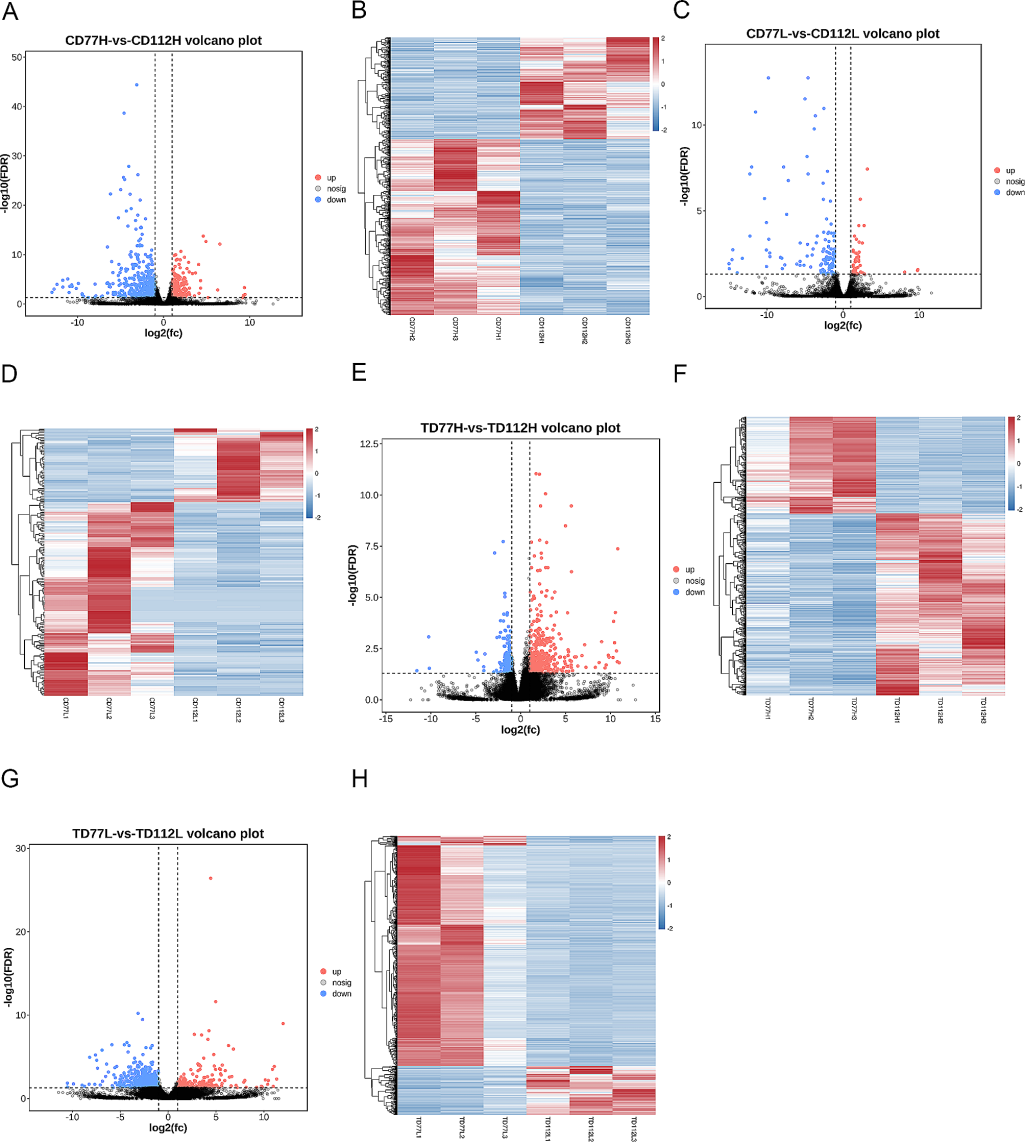
DEGs in the comb and testis tissues of the high-or low-comb height groups at different developmental stages. (**A** and **B**) The volcano map (**A**) and heat map (**B**) of the DEGs in the comb tissues of the H group at 77 and 112 days of age; (**C** and **D**) the volcano map (**C**) and heat map (**D**) of the DEGs in the comb tissues of the L group at 77 and 112 days of age; (**E** and **F**) the volcano map (**E**) and heat map (**F**) of DEGs in the testes tissues of the H group at 77 and 112 days of age; and (**G** and **H**) the volcano maps (**G**) and heat map (**H**) of the DEGs in the testes tissues of the L group at 77 and 112 days of age

We conducted DEGs analysis of the comb and testis tissues of the H or L group roosters at 77 and 112 days old. We identified a total of 869 and 1060 DEGs in the comb (332 upregulated and 537 downregulated genes; 4 A and 4B) and testis (492 upregulated and 568 downregulated genes; Fig. 4E and F) tissues of the H group at 77 and 112 days of age, respectively. In contrast, we identified 199 and 745 DEGs in the comb (53 upregulated and 146 downregulated genes; 4 C and 4D) and testis (167 upregulated and 578 downregulated genes; Fig. 4G and H) tissues of the L group at 77 and 112 days of age, respectively.

**Fig. 5:**
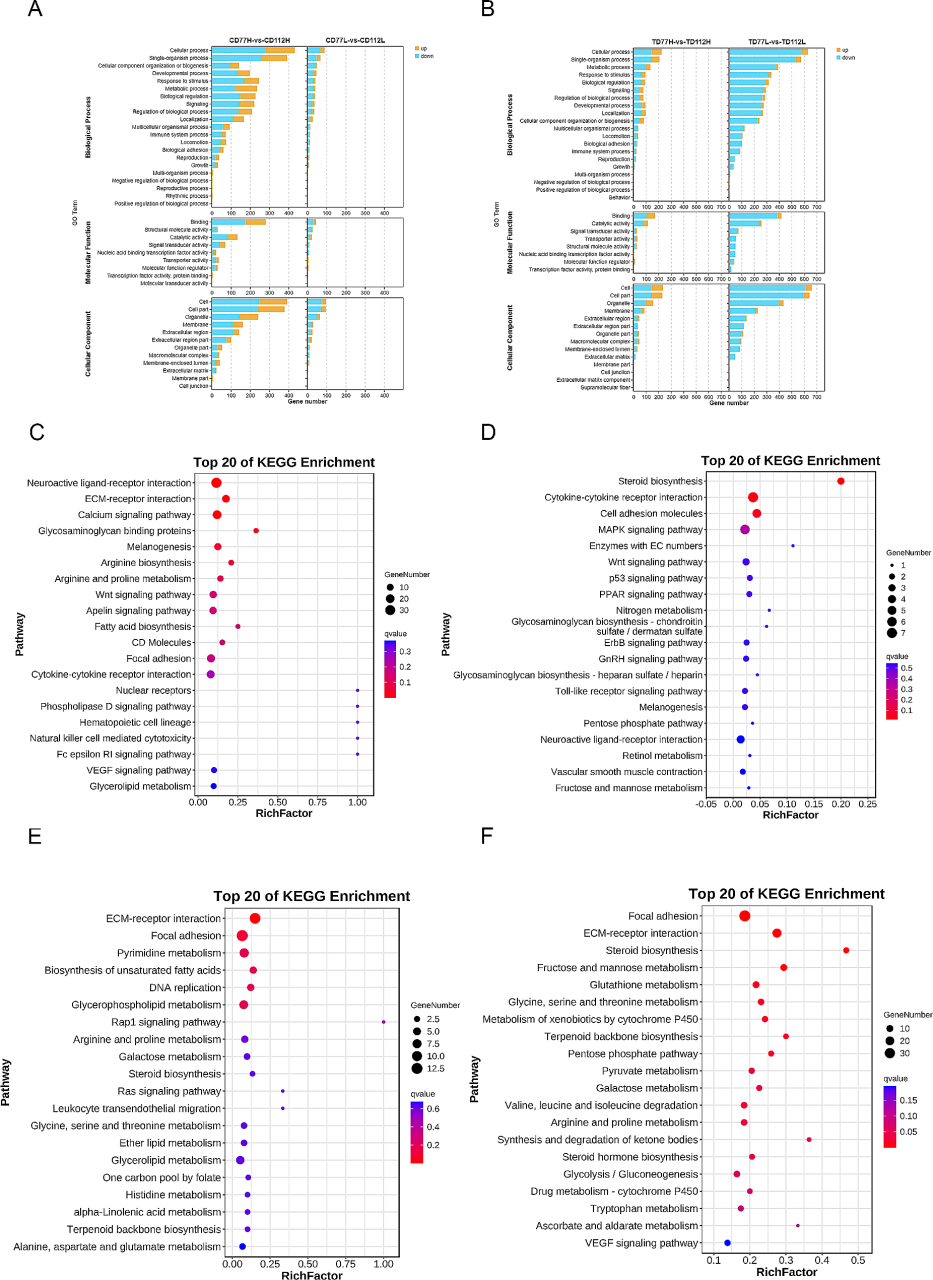
GO and KEGG analysis of DEGs in the comb and testis tissues of the high- or low-comb groups at different developmental stages. (**A** and **B**) GO enrichment analysis of the DEGs in the comb (**A**) and testes (**B**) tissues in the molecular function, cellular composition, and biological processes categories; (**C** and **D**) KEGG enrichment analysis of the DEGs in the comb tissues of the high- (**C**) and low-comb groups (**D**) at 77 and 112 days of age; (**E** and **F**) KEGG enrichment analysis of the DEGs in the testes tissues of the high- (**E**) and low-comb groups (**F**) at 77 and 112 days of age

These DEGs were subjected to GO and KEGG enrichment analyses to determine their biological functions. GO enrichment analysis showed that these DEGs were also significantly enriched in signal-organism processes, cellular processes, response to stimulus, and developmental processes (Fig. 5A and B). KEGG enrichment analysis showed that the DEGs in the comb tissues of the H group at 77 and 112 days of age were significantly enriched in glycosaminoglycan binding proteins, calcium signaling pathway, and VEGF signaling pathway (Fig. 5C) and that the DEGs in the testis tissues of the H group at 77 and 112 days of age were significantly enriched in ECM-receptor interaction, focal adhesion, and biosynthesis of unsaturated fatty acid (Fig. 5E). Additionally, KEGG enrichment analysis showed that the DEGs in the comb tissues of the L group at 77 and 112 days of age were significantly enriched in steroid biosynthesis, cytokine-cytokine receptor interaction, MAPK signaling pathway and cell adhesion molecules pathway (Fig. 5D) and that the DEGs in the testis tissues of the L group at 77 and 112 days of age were significantly enriched in focal adhesion and the VEGF signaling pathway (Fig. 5F). 

We conducted a comparative analysis of the DEGs in the comb and testis tissues of the H or L group at 77 and 112 days of age and identified 33 DEGs in the comb and testis tissues of the H group. Among these, WNT1 inducible signaling pathway protein 2 (*WISP2*), peptidase domain containing associated with muscle regeneration 1 (*PAMR1*), interleukin 17 receptor E like (*IL17REL*), interleukin-13 receptor subunit alpha-2 precursor (*IL13RA2*), gliomedin (*GLDN*), myelin proteolipid protein (*PLP1*), galactose mutarotase (*GALM*), fibromodulin precursor (*FMOD*), sarcoglycan alpha (*SGCA*), actin gamma-enteric smooth muscle (*ACTG2*), and desmin (*DES*) were significantly downregulated and may be associated with the regulation of the precocious phenotype of the comb and testis tissues in the Qingyuan Partridge roosters. Additionally, we found 15 DEGs in the comb and testis tissues of the L groups, among which NDRG family member 4 (*NDRG4*), *WNT6*, and *AR* may be responsible for the regulation of the compensatory growth during the late developmental stages of the sex late-maturing in Qingyuan Partridge roosters.

### WGCNA of the DEGs in the comb and testis tissues

**Fig. 6:**
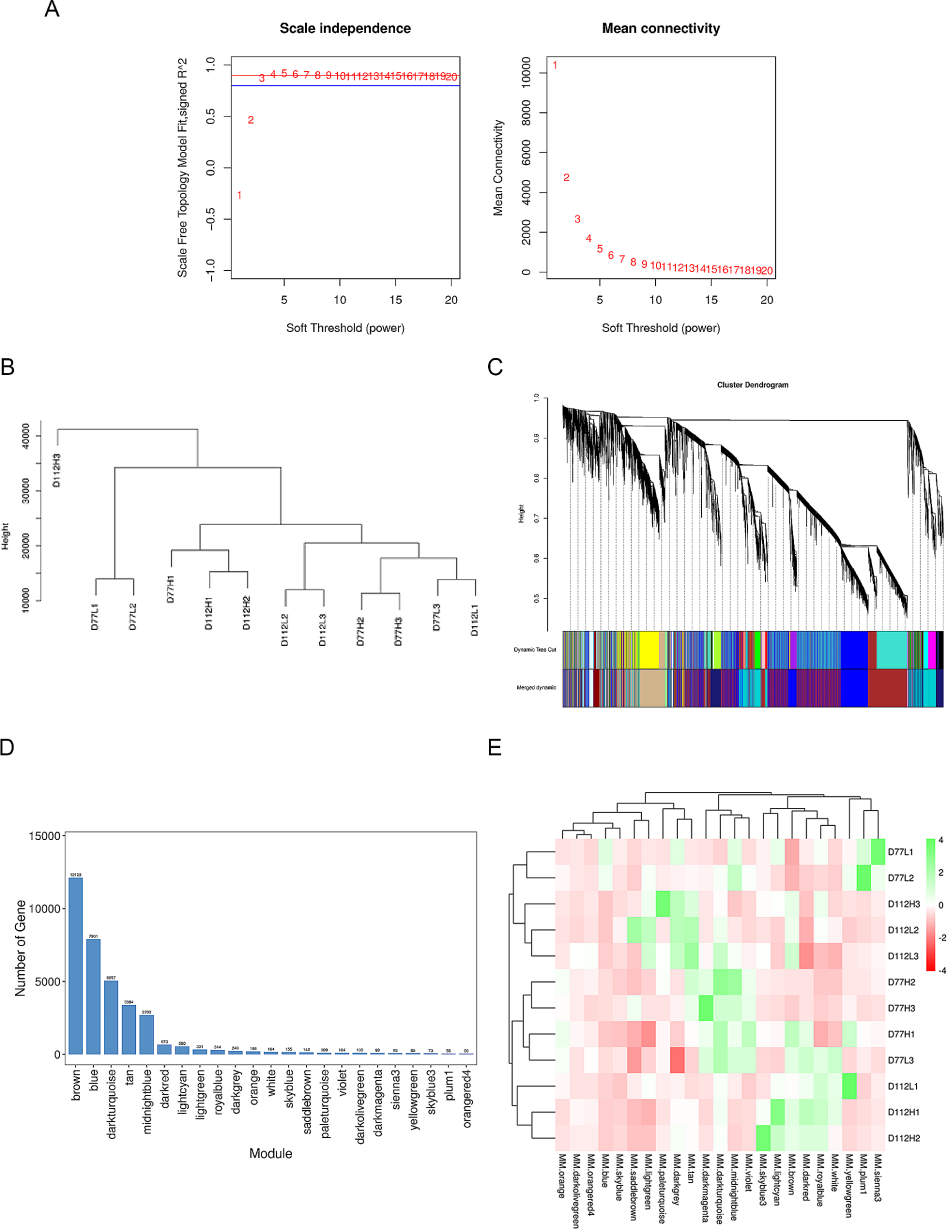
Weighted correlation network analysis of the DEGs in the comb and testes tissues. (**A**) Analysis of the scale-free fitting index of various soft-thresholding powers (left) and the average connectivity of various soft-thresholding powers (right); (**B**) sample tree obtained from hierarchical clustering; (**C**) gene dynamic shearing clustering tree, each color represents a module; (**D**) classification of the DEGs into 21 modules according to the gene co-expression patterns; (**E**) the heat map of gene expression patterns of different phenotypic tissues at different developmental stages

To further explore the genes related to the difference of sexual maturity in different growth stages of Qingyuan Partridge roosters, we conducted a WGCNA of the DEGs in the comb and testis tissues of the H and L groups at different developmental stages. When the scale-free topology model fit (R^2^) reached 0.8, the soft thresholding power (β) was 4 (Fig. 6A). Hierarchical clustering among all the tissues was presented as an array tree (Fig. 6B). A total of 34,700 DEGs were clustered into 21 modules (Fig. 6C), among which, the brown module contained the highest number of genes (12,123 genes), while the orangered4 module contained the lowest number of genes (55 genes; Fig. 6D). A sample expression model heat map was used to compare the modules with similar expression coefficients for different traits at different ages, and the blue, darkolivegreen, sienna3, and plum1 modules were used to represent the 77-day-old H group, 77-day-old L group, 112-day-old H group, and 112-day-old L group, respectively (Fig. 6E).

**Fig. 7:**
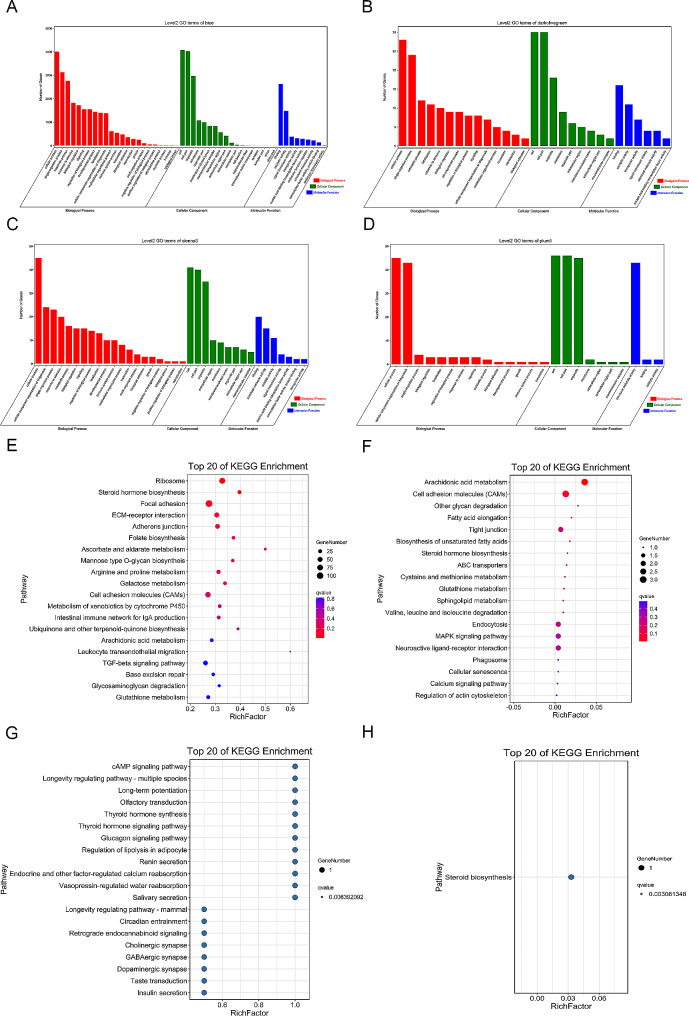
GO and KEGG analysis of the high-expression modules. (**A**-**D**) GO enrichment analysis of the blue (**A**), darkolivegreen (**B**), sienna3 (**C**), and plum1 (**D**) modules in the molecular function, cellular composition, and biological processes categories; (**E**-**H**) KEGG enrichment analysis of the blue (**E**), darkolivegreen (**F**), sienna3 (**G**), and plum1 (**H**) modules

GO enrichment analysis showed that the DEGs in the blue module were significantly enriched in cellular processes, cell part, and signal-organism processes (Fig. 7A); DEGs in the darkolivegreen module were significantly enriched in cell and cell part (Fig. 7B); DEGs in the sienna3 module were significantly enriched in organelle and cellular component organization or biogenesis (Fig. 7C); and DEGs in the plum1 module were significantly enriched in organelle and structural molecule activity (Fig. 7D). Furthermore, we found that 36 GO pathways were enriched at both the developmental stages in the combs of the H group, while 14 GO pathways were enriched only at 77 days of age. Moreover, we found that 22 GO pathways were enriched at both the developmental stages in the combs of the L group, while 7 GO pathways were enriched only at 77 days of age and 2 GO pathways were enriched only at 112 days of age (Table S2). Further analysis of the DEGs revealed that *SFRP1*, *WNT6*, *KPNA7*, and *CCN3*, associated with gonadal, epidermal, and vascular development, may play a role in regulating precocious and delayed puberty in Qingyuan Partridge roosters.

KEGG enrichment analysis showed that the DEGs in the blue module were significantly enriched in ribosome, steroid hormone biosynthesis, and focal adhesion pathway (Fig. 7E); DEGs in the darkolivegreen module were significantly enriched in arachidonic acid metabolism and cell adhesion molecules (Fig. 7F); DEGs in the Sienna3 module were significantly enriched in cAMP signaling pathway, longevity regulating pathway-multiple species, and long-term potentiation (Fig. 7G); and genes in the plum1 module were significantly enriched in steroid biosynthesis (Fig. 7H). We further found that 13 KEGG pathways were enriched only at 77 days of age in the H group, while 3 KEGG pathways were enriched only at 77 days of age and 1 KEGG pathway was enriched only at 112 days of age in the L group (Table S3). These results suggest that the pathways enriched only in the H group at 77 days of age may be responsible for the differences in the comb height phenotypes between precocious and delayed puberty in Qingyuan Partridge roosters, while the pathways enriched only in the L group at 112 days of age may be responsible for regulating the compensatory development in delayed puberty Qingyuan Partridge roosters.

**Fig. 8:**
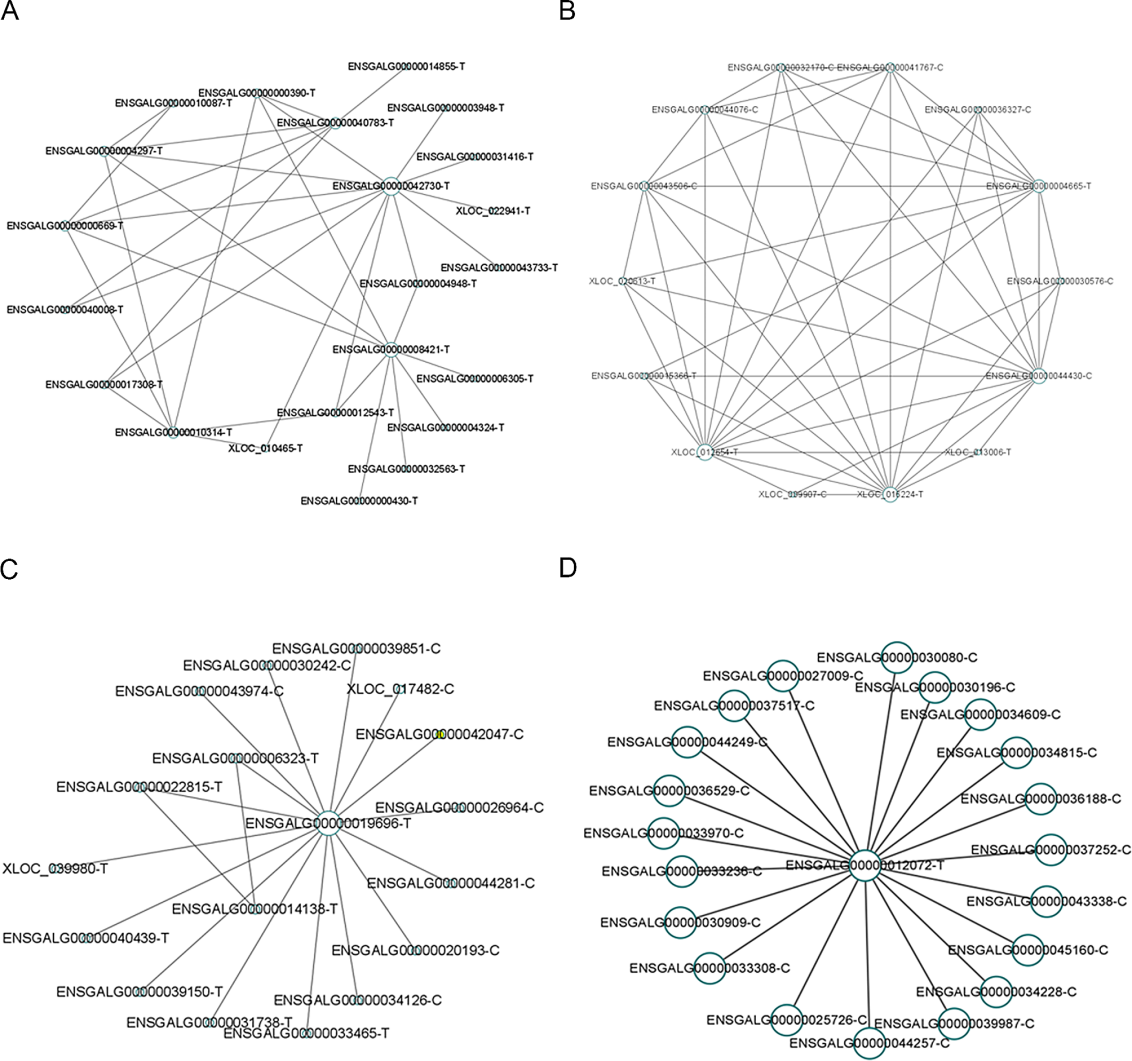
Construction of the co-expression subnetwork and screening of the hub genes. (**A**-**D**) Gene co-expression network of the blue (**A**), darkolivegreen (**B**), sienna3 (**C**), and plum1 (**D**) modules

Cytoscape was used to construct gene interaction networks of the four modules, and Cytohubba analysis found that STT3 oligosaccharyltransferase complex catalytic subunit A (*STT3A*) and peroxisomal biogenesis factor 1(*PEX1*) were the hub genes in the blue module, while karyopherin subunit alpha 7 (*KPNA7*), *ATHL*, and *ENSGALG00000012072* were the hub genes in the darkolivegreen, sienna3, and plum1 modules, respectively (Fig. 8A and D). In addition, previously discovered *WNT6*, *AMH*, *IHH*, *ROR2*, *SFRP1*, *CYP26B1*, *PAMR1*, *IL17REL*, *WISP2*, *NDRG4*, and *HTRA1* were identified as the hub genes in the blue module, while *CRHBP* was identified as the hub gene in the sienna3 module. Altogether, these results reveal that these hub genes may be responsible for regulating the sexual maturity of Qingyuan Partridge roosters, via pathways associated with blood vessels, skin connective tissue development, energy storage, and cell growth.

## Discussion

Comb height is a heritable trait [16], and it is usually used as the main reference trait in the selection of sexual precocity in roosters. Moreover, comb height can be used as an important indicator of sexual maturity and testis development [10]. In this study, we found that capillary dilation and loosening of collagen fiber arrangement occurred earlier in the H group compared to the L group at 56 and 77 days of age, consistent with the results observed by Yoshioka [24]. The density difference of collagen fibers may be the primary reason for the high and low phenotypes of the combs. However, the morphological changes in the comb tissues of the H and L groups converged gradually at 112 days of age, although the underlying mechanism is unknown and needs to be further investigated. Additionally, we found that the sexual maturation of the L group was delayed compared to that of the H group during early developmental stages; however, the L group showed compensatory growth during the late developmental stage. In our previous study, we found that the body weight of the low-comb roosters was higher than that of the high-comb roosters, during the later stages of growth and development. Moreover, studies have shown that slow-growing chickens contain higher levels of inosinic acid, suggesting that their flavor may be better than that of fast-growing chickens [25]. Therefore, further studies can be conducted on the breeding of low-comb roosters into a heavier and more flavorful late-maturing strain.

In poultry industry, the reproductive performance of roosters is crucially important [26]. In recent years, a multitude of scholars have conducted extensive research on the genetic mechanism underlying the process of sexual maturation in chickens. Previous studies have reported *WNT6* and *AMH* as key genes associated with gonadal development in chickens [16, 27–29]. Similarly, the present study reveals that *WNT6* exhibits the potential to impede the development of comb and testis in the H group during the initial stages of development, whereas *AMH* demonstrates the capacity to foster the advancement of comb and testis development in the L group during the later stages of development. The DEGs analysis of the RNA-seq data further demonstrated that the expression levels of genes *WISP2*, *PAMR1*, and *IL17REL* were significantly reduced in the H group, suggesting their potential role in regulating early maturation traits in roosters. Conversely, genes *CRHBP, CYP26B1*and *NDRG4* exhibited differential expression exclusively in the L group, possibly indicating their involvement in regulating compensatory testicular and comb growth during the late developmental stages in the L group. *WISP2* exhibits significant expression levels within the reproductive tissues and has been demonstrated to play a role in the modulation of avian development, cellular adhesion and proliferation, as well as the remodeling of the extracellular matrix [30]. Although the role of *PAMR1* and *IL17REL* in chicken growth and development remains to be investigated, it is speculated that the downregulation of *WISP2*, *PAMR1*, and *IL17REL* genes observed in H group may indicate their inhibitory effects on comb and testicular development.

CYP26B1 is a retinoic acid-degrading enzyme that can regulate the initiation of meiosis in chicken germ cells [31]. *NDRG4* is a downstream regulatory gene of N-myc and is involved in the regulation of cell proliferation, differentiation, and tissue development in chickens [32]. Therefore, it is postulated that the differentially expressed *CYP26B1* and *NDRG4* genes, exclusively observed in the L group, potentially participate in governing the compensatory growth of the L group’s testes and combs during the later phases of development by modulating cellular proliferation and tissue development.

GO and KEGG analysis revealed that the DEGs were primarily enriched in MAPK signaling, VEGF signaling, and retinol metabolism pathways. MAPK signaling is a common reproductive-related signaling pathway and is associated with angiogenesis and chondrocyte development [33]. Moreover, some studies have shown that long non-coding RNAs regulate the formation of primordial germ cells by activating the MAPK signaling pathway in chickens [34]. The VEGF signaling pathway is crucial for angiogenesis, development, and maintenance of physiological homeostasis [35]. Lastly, the retinol metabolic pathway plays an important role in the differentiation of male germ cells in chickens [36]. The presence of enriched DEGs within these pathways may account for the observed early onset of capillary anastomosis, loosening of the collagen fiber network, and intact structure of testicular seminiferous tubules in H-group roosters as compared to L-group roosters. Therefore, these DEGs can be considered potential candidate genes for the regulation of precocious and delayed puberty in Qingyuan Partridge roosters.

WGCNA has been shown to play an important role in screening key genes and signaling pathways [37, 38]. To our knowledge, this is the first study to use WGCNA to screen candidate genes that affect the development of comb and testis tissues of Qingyuan Partridge roosters. WGCNA of the DEGs identified four hub genes, including *STT3A*, *PEX16*, *KPNA7*, and *CATHL2*, associated with comb and testis development in the roosters. *STT3A* is involved in the regulation of glycosylation, which may affect the development and differentiation of cells and tissues [39, 40]. *PEX16* plays a key role in the development of white adipocytes and lipid homeostasis, which may regulate energy storage during the development of sexual maturity in roosters [41]. *KPNA7* is associated with cell mitosis, and its deletion can significantly reduce cell growth [42]. It is postulated that these core genes may exert an impact on sexual maturation via their regulatory roles in protein post-translational modification, adipogenesis, or cellular proliferation. *CATHL2* has been reported to be associated with innate immunity in chickens, with the downregulation of *CATHL2* leading to a decrease in innate immune function in chickens [43]. However, additional research is required to investigate its involvement in the regulation of sexual maturity. Moreover, we found additional hub genes (including *IHH* and *ROR2*) that were associated with comb and testis development in roosters. *IHH* plays a crucial role in regulating the proliferation and hypertrophy of chicken chondrocytes [44]. *ROR2* is a signaling receptor for Wnt ligands and plays an important role in limb development [45]; additionally, it has been reported as a potential candidate gene for testicular growth and development in chickens [46]. The candidate genes associated with chicken chondrogenesis have the potential to impact the formation of dense connective tissue, consequently resulting in the accelerated onset of early sexual maturity in Qingyuan Partridge roosters.

## Conclusion

In summary, these results provide a comprehensive transcriptomic analysis of the combs and testes of Qingyuan Partridge roosters at the age of 77 and 112 days. WGCNA was used to identify the candidate genes (*WNT6*, *AMH*, *IHH*, *STT3A*, *PEX16*, *KPNA7*, *CATHL2*, *ROR2*, *PAMR1*, *WISP2*, *IL17REL*, *NDRG4*, *CYP26B1*, and *CRHBP*) that affect precocious and delayed puberty in Qingyuan Partridge roosters. Therefore, our findings provide the basis for understanding the underlying molecular mechanism associated with sexual maturity and its related traits in chickens.

## Materials and methods

### Animals and tissues

Qingyuan Partridge roosters were obtained from the Original Breeding Farm of Guangdong Tiannong Food Co., Ltd. (Guangdong, China). The management procedures were conducted according to the standard requirements for the chicken breeding farm. The lighting conditions have equal periods of bright and dark. The roosters (42 days old) were divided into high-comb (H group; 22–29 mm, n = 260) and low-comb (L group; 10–17 mm, n = 210) groups, according to the comb height. The comb height difference between the two groups is 9.97 mm, which indicates a significant distinction.15 roosters were slaughtered from each group on days 56 (the rapid growth period of Qingyuan Partridge roosters), 77 (the early growth and development period), and 112 (the later stages of development), and comb and testicular tissues were collected. The tissues were stored in a 4% paraformaldehyde solution for histological analysis or frozen in liquid nitrogen and stored at − 80 ℃ for RNA extraction.

### Determination of body weight and coefficients of testis

The pertinent characteristics of roosters at the ages of 56, 77, and 112 days were assessed utilizing an electronic scale, and subsequently computed in the following manner. Body weight was measured after roosters fasted for ~ 12 h. The comb height is determined by employing a vernier caliper to precisely measure the vertical extent between the uppermost comb tip and the base of the comb. Testicular weight was measured by extracting both left and right testicles (excluding the epididymis) in their entirety after dissecting the carcass. The long and short diameters of each testicle were measured using a vernier caliper. The long diameter was determined as the linear distance between the upper and lower extremities of the testicle, whereas the short diameter was determined as the linear distance between the anterior and posterior edges of the testicle. Subsequently, the testicular area was calculated as (short diameter×long diameter). The calculation for the testis index is as follows: Testis index (%) = Testis weight(mg)/body weight(g) × 100. All measured values are expressed as mean ± SD.

### Histological analysis

Tissues for histological analysis were prepared as previously described [47]. The comb and testis tissues were fixed in 4% paraformaldehyde solution for 10 d. Thereafter, the tissues were paraffin-embedded, sectioned (5–7 μm), hematoxylin-eosin (HE) stained, and observed under a 400X optical microscope (Leica, Heidelberg, Germany).

### RNA extraction and sequencing

The total RNA was extracted from the comb and testes tissues using the Total RNA Extraction Kit (Sangong, Shanghai, China) according to the manufacturer’s instructions. The concentration and quality of all the RNA tissues were determined using a micro nucleic acid/protein concentration tester (Eppendorf, Hamburg, Germany). Subsequently, equal amounts of total RNA of each group were pooled for comb and testes tissues and used to construct the libraries for transcriptome analysis. RNA-seq of these tissues was conducted by Genedenovo Biotech (Guangzhou, China). FASTP was used for quality control processing to filter the low-quality data, and HISAT2 was used to align clean data to the chicken reference genome. DESeq2 was used to analyze differential expression among the tissues at FDR < 0.05 and|log2FC| >1. The Gene Ontology (GO) and Kyoto Encyclopedia of Genes and Genomes (KEGG) enrichment analyses were performed to determine the biological functions of the differentially expressed genes (DEGs).

### Construction of co-expression network

Two tissues from the same developmental stage were pooled for the weighted correlation network analysis (WGCNA). The co-expression network was constructed for the mRNA expression profile data using the R package, WGCNA. The sample cluster tree was established to filter the outliers. The adjacency matrix was converted to a topological overlap matrix to reduce the effects of spurious associations and noise. Thereafter, the dynamic tree cut algorithm was used to identify the gene co-expression modules, with each module containing at least 50 genes. Finally, highly similar modules were merged and represented with different colors (merge cut height = 0.25), and module membership and gene significance were calculated to identify hub genes.

### Real-time quantitative PCR validation of candidate genes

Real-time quantitative PCR (RT-qPCR) was used to detect the mRNA expression of the target genes. Total RNA from comb and testis tissues was reverse transcribed using the Evo M-MLV Reverse Transcriptase Kit (Ruizhen, Guangzhou, China) according to the manufacturer’s instructions. The qPCR reactions were performed using the AceQ qPCR SYBR Green Master Mix (Vazyme, Nanjing, China) on the CFX96 Touch RT-qPCR System (Bio-Rad, California, USA). All the primers (Table S1) used in this study were designed using the Primer Premier 5.0 software (Premier Biosoft, Palo Alto, CA, USA). The *GAPDH* mRNA was used as an internal control, and the relative mRNA expression was determined using the 2^-∆∆Ct^ method.

### Statistical analysis

Statistical data analysis in this study was performed using the SPSS 20.0 software package (IBM). The t-test and one-way analysis of variance (ANOVA) were used appropriately to test the result of the comparison. Statistical significance was declared at *p* < 0.05.

### Electronic supplementary material

Below is the link to the electronic supplementary material.

Additional file 1: Figure S1: Real-time quantitative PCR (RT-qPCR) analysis of the candidate DEGs

Additional file 2: Figure S2: Heat map of the DEGs in the comb and testes tissues of the high- and low-comb groups at the same developmental stage

Additional file 3: Table S1: Primers for RT-qPCR analysis

Additional file 4: Table S2: GO pathway was expressed only in single-day traits

Additional file 5: Table S3: KEGG pathways were expressed only in single-day traits

## Data Availability

The raw data of mRNA sequencing has been submitted to the NCBI Sequence Read Archive (SRA) with the accession number PRJNA1013206.
