# Supplementary figures and images for: Analysis of the differentially expressed genes in the combs and testes of Qingyuan partridge roosters at different developmental stages
Source: BMC Genomics. 2024 Jan 4;25:33. doi: 10.1186/s12864-024-09960-2 (PMC10768254; doi:10.1186/s12864-024-09960-2)

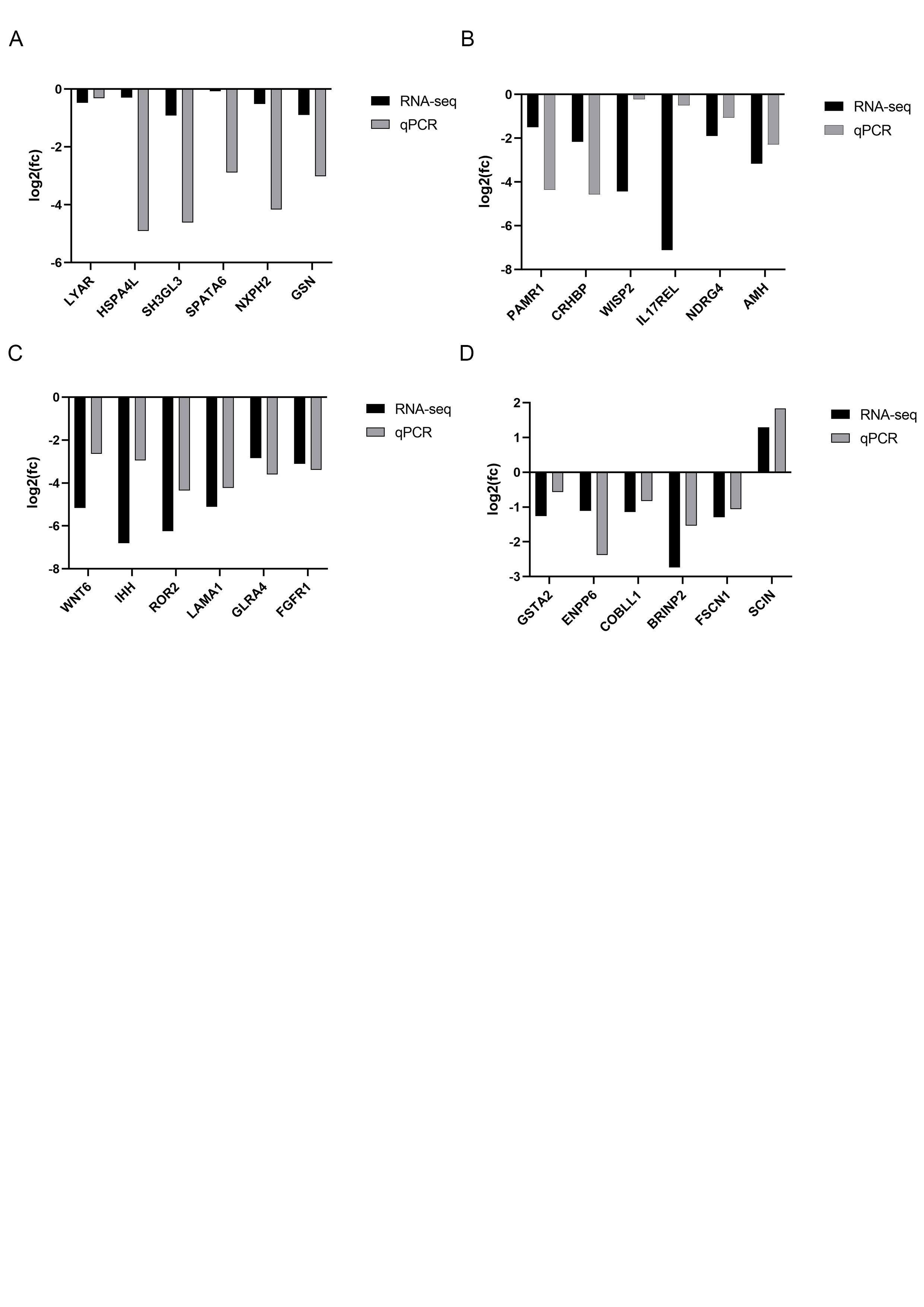

Supplement: Supplementary file 1 — Additional file 1: Figure S1: Real-time quantitative PCR (RT-qPCR) analysis of the candidate DEGs [file 12864_2024_9960_MOESM1_ESM.png]

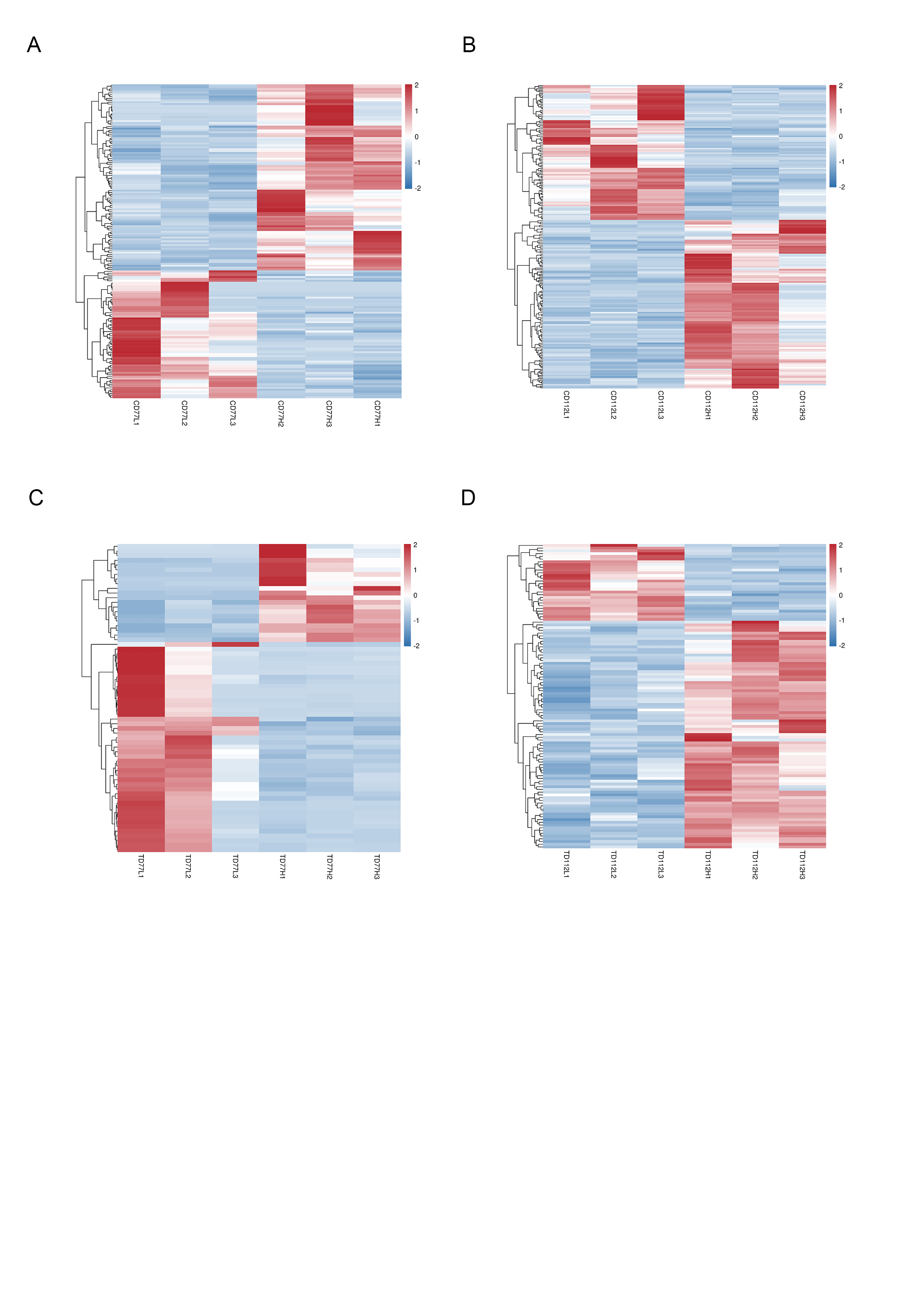

Supplement: Supplementary file 2 — Additional file 2: Figure S2: Heat map of the DEGs in the comb and testes tissues of the high- and low-comb groups at the same developmental stage [file 12864_2024_9960_MOESM2_ESM.png]
