# Supplementary material for: Analysis of the differentially expressed genes in the combs and testes of Qingyuan partridge roosters at different developmental stages
Source: BMC Genomics. 2024 Jan 4;25:33. doi: 10.1186/s12864-024-09960-2 (PMC10768254; doi:10.1186/s12864-024-09960-2)
Supplement: Supplementary file 3 — Additional file 3: Table S1: Primers for RT-qPCR analysis [file 12864_2024_9960_MOESM3_ESM.docx]

Table S1 Primers for RT-qPCR analysis

| Gene | Accession No. | Primer sequence (5'-3') | annealing temperature |
| --- | --- | --- | --- |
| *LYAR* | XM_420792.8 | F: 5'-TGGACTGTGGGAAGGATTTC-3'  R: 5'-GAACGCATTTGCTCCAGAAT-3' | 58℃ |
| *HSPA4L* | NM_001012576.2 | F: 5'-AGCTGCATGGAAGAGCATTT-3'  R: 5'-GCCACTGGTTTCTTCAGAGC-3' | 58℃ |
| *SH3GL3* | NM_204528.2 | F: 5'-AAGCCAGCCAGTTGTTCAGT-3'  R: 5'-TTCCCAGCTTGGCTCTGTAT-3' | 58℃ |
| *SPATA6* | XM_040705414.2 | F: 5'-CAGCGCCTAGTGTTTGATGA-3'  R: 5'-CTCTGTGAGGCTTCCCTTTG-3' | 58℃ |
| *NXPH2* | XM_004943002.5 | F: 5'-GACGGCTCAGAAGGACTCAC-3'  R: 5'-GTTAGGTTCCTGGCGAAACA-3' | 58℃ |
| *GSN* | NM_204934.2 | F: 5'-GGGCAGGAGAACAGTCAGAG-3'  R: 5'-GCTAGGACAGTGGCCTTCAG-3' | 58℃ |
| *WNT6* | NM_001007594.3 | F: 5'-GACGTGCAGTTTGGCTATGA-3'  R: 5'-GCATTTGCACTCTGTCCTCA-3' | 60 ℃ |
| *AMH* | NM_205030.2 | F: 5'-AGGAAGTGAAGTGGGAAGCA-3'  R: 5'-GGGTGTCCCTAGTGAAGCAA-3' | 60 ℃ |
| *IHH* | NM_204957.3 | F: 5'-CCAACTACAACCCCGACATC-3'  R: 5'-GTACTTGTTGCGGTCCCTGT-3' | 60 ℃ |
| *ROR2* | NM_204957.3 | F: 5'-GGAATTGTGCAGGGATGAGT-3'  R: 5'-TTTCGTGACGCTGACTGTTC-3' | 60 ℃ |
| *LAMA1* | NM_001199806.2 | F: 5'-CGATCATCCGTGCTATCCTT-3'  R: 5'-TGCACAACGGTCACATTTTT-3' | 60 ℃ |
| *GLRA4* | XM_015278390.4 | F: 5'-CGGATTTGTTCTTTGCCAAT-3'  R: 5'-AGGTCGTTCATGGTGTAGCC-3' | 60 ℃ |
| *FGFR1* | NM_205510.2 | F: 5'-GTCTCAGACGCACTCCCTTC-3'  R: 5'-GTCAGGCTTGAACTCCTTGC-3' | 60 ℃ |
| *PAMR1* | XM_421084.8 | F: 5'-AATCTGCGGAAAAGCAGAAA-3'  R: 5'-TCTTGAGCACGATGGTCTTG-3' | 60 ℃ |
| *WISP2* | XM_417370.8 | F: 5'-TCCAACCAGAACCCCTACTG-3'  R: 5'-TGAAGTCCATCGGTTCTTCC-3' | 60 ℃ |
| *IL17REL* | XM_015273696.4 | F: 5'-TCCAGATGCCAGGAGGTTAC-3'  R: 5'-TGCAAAGTCTTGGGTGAGTG-3' | 60 ℃ |
| *NDRG4* | XM_004944013.5 | F: 5'-ATGTGATTGGGATCGGTGTT-3'  R: 5'-TGCCTGTAACTCTGCACCAG-3' | 60 ℃ |
| *CRHBP* | XM_025145048.3 | F: 5'-ACCTCTGCTGAGTTCGCAAT-3'  R: 5'-GCAGCTTTCGCTGGAATTAG-3' | 60 ℃ |
| *GSTA2* | NM_001001776.2 | F: 5'-TAAAGGCTGTTGGACCTGCT-3'  R: 5'-GGAAGAGGCTTTGTTTGCAG-3' | 58℃ |
| *ENPP6* | XM_420512.8 | F: 5'-ATCATCCGCTCGCAATTAAC-3'  R: 5'-AGGGTGAGGAAGACGAGGAT-3' | 58℃ |
| *COBLL1* | XM_040703572.2 | F: 5'-GGGAATGCTTGAAGTGGAAA-3'  R: 5'-CAAGGGCTTCTTGAGACTGG-3' | 60 ℃ |
| Gene |  | Primer sequence (5'-3') |  |
| *BRINP2* | XM_025153140.3 | F: 5'-CCTGAACAGGACAGCCATCT-3'  R: 5'-CTCCTTTGGCAGTTTGAAGC-3' | 60 ℃ |
| *FSCN1* | NM_001178132.2 | F: 5'-GGCAAATACCTGAAGGGTGA-3'  R: 5'-CCAGGCTGGAAATCCTTGTA-3' | 60 ℃ |
| *SCIN* | NM_001244593.2 | F: 5'-CCAGGATTGCTGAGGTTGAT-3'  R: 5'-CTGGTTCCTGGCCTTCATTA-3' | 60 ℃ |
| *GAPDH* | NM_204305.2 | F: 5'-GAACATCATCCCAGCGTCCA-3'  R: 5'-ACGGCAGGTCAGGTCAACAA-3' | 60 ℃ |
