# Supplementary material for: Analysis of the differentially expressed genes in the combs and testes of Qingyuan partridge roosters at different developmental stages
Source: BMC Genomics. 2024 Jan 4;25:33. doi: 10.1186/s12864-024-09960-2 (PMC10768254; doi:10.1186/s12864-024-09960-2)
Supplement: Supplementary file 4 — Additional file 4: Table S2: GO pathway was expressed only in single-day traits [file 12864_2024_9960_MOESM4_ESM.docx]

Table S2 GO pathway was expressed only in single-day traits

| Group | 77-day-old high comb group | 77-day-old low comb group | 112-day-old low comb group |
| --- | --- | --- | --- |
| GO pathway | cell junction  (GO:0030054) | reproduction  (GO:0000003) | immune system process  (GO:0002376) |
|  | reproductive process  (GO:0022414) | organelle part  (GO:0044422) | growth  (GO:0040007) |
|  | virion  (GO:0019012) | signal transducer activity  (GO:0004871) |  |
|  | supramolecular fiber  (GO:0099512) | multicellular organismal process  (GO:0032501) |  |
|  | extracellular matrix component  (GO:0044420) | membrane-enclosed lumen  (GO:0031974) |  |
|  | molecular function regulator  (GO:0098772) | nucleic acid binding transcription factor activity  (GO:0001071) |  |
|  | behavior  (GO:0007610) | transporter activity  (GO:0005215) |  |
|  | membrane part  (GO:0044425) |  |  |
|  | virion part  (GO:0044423) |  |  |
|  | synapse part  (GO:0044456) |  |  |
|  | synapse  (GO:0045202) |  |  |
|  | molecular transducer activity  (GO:0060089) |  |  |
| Group | 77-day-old high comb group | 77-day-old low comb group | 112-day-old low comb group |
| GO pathway | extracellular matrix  (GO:0031012) |  |  |
|  | rhythmic process  (GO:0048511) |  |  |
