# Supplementary material for: Analysis of the differentially expressed genes in the combs and testes of Qingyuan partridge roosters at different developmental stages
Source: BMC Genomics. 2024 Jan 4;25:33. doi: 10.1186/s12864-024-09960-2 (PMC10768254; doi:10.1186/s12864-024-09960-2)
Supplement: Supplementary file 5 — Additional file 5: Table S3: KEGG pathways were expressed only in single-day traits [file 12864_2024_9960_MOESM5_ESM.docx]

Table S3 KEGG pathways were expressed only in single-day traits

| Group | 77-day-old high comb group | 77-day-old low comb group | 112-day-old low comb group |
| --- | --- | --- | --- |
| KEGG pathway | ribosome  (ko03010) | arachidonic acid metabolism  (ko00590) | steroid biosynthesis  (ko00100) |
|  | steroid hormone biosynthesis  (ko00140) | cell adhesion molecules  (ko04514) |  |
|  | focal adhesion  (ko04510) | other glycan degradation  (ko00511) |  |
|  | ECM-receptor interaction  (ko04512) |  |  |
|  | adherens junction  (ko04520) |  |  |
|  | folate biosynthesis  (ko00790) |  |  |
|  | ascorbate and aldarate metabolism  (ko00053) |  |  |
|  | mannose type O-glycan biosyntheis  (ko00515) |  |  |
|  | arginine and proline metabolism  (ko00330) |  |  |
|  | galactose metabolism  (ko00052) |  |  |
|  | metabolism of xenobiotics by cytochrome P450  (ko00980) |  |  |
|  | intestinal immune network for IgA production  (ko04672) |  |  |
|  | ubiquinone and other terpenoid-quinone biosynthesis  (ko00130) |  |  |
